# A Study of Methadone-Poisoned Children Referred to Hamadan’s Besat Hospital/Iran

**Published:** 2014

**Authors:** Hassan BAZMAMOUN, Afshin FAYYAZi, Ali KHAJEH, Mohammad Kazem SABZEHEI, Fuzieh KHEZRIAN

**Affiliations:** 1 Department of Pediatric Gastroenterology, Hamadan University of Medical Sciences, Hamadan, Iran; 2 Pediatric Neurology Department, Hamadan University of Medical Sciences, Hamadan, Iran; 3 Pediatric Neurology Department, Zahedan University of Medical Sciences, Zahedan, Iran; 4 Department of Neonatology, Hamadan University of Medical Sciences, Hamadan, Iran; 5 Hamadan University of Medical Sciences, Hamadan, Iran

**Keywords:** Methadone, Poisoning, Children, Prognosis

## Abstract

**Objective:**

Increasing use of methadone in withdrawal programs has increased methadone poisoning in children. This research aimed to study the causes of incidence of poisoning in children and its side-effects.

**Materials & Methods:**

In this research, The hospital records of all methadone-poisoned children referred to Hamadan’s Be’sat Hospital from June 2007 to March 2013, were studied. Children with a definite history of methadone use or proven existence of methadone in their urine, were studied.

**Results:**

During 5 years, 62 children with the mean age of 53.24±29.50 months were hospitalized due to methadone use. There was a significant relationship between delayed referral to hospital and increased bradypnea. According to their history, 25.8% and 58.1% of the children had been poisoned by methadone tablet and syrup, respectively. The most common initial complaint expressed by parents, was decreased consciousness (85.5%). During the initial examination, decreased consciousness, meiosis, and respiratory depression were observed in 91.9%, 82.3%, and 69.4% of the cases, respectively. Nine patients required mechanical ventilation. There was a significant relationship between the need for mechanical ventilation and seizure with initial symptom of emesis. There were two cases of death (3.2%), both of which were secondary to prolonged hypoxia and brain death. There was a significant relationship between poor patient prognosis (death) and presence of cyanosis in early symptoms, seizure, hypotension, duration of decreased consciousness, and duration of mechanical ventilation.

**Conclusion:**

This research indicated that the occurrence of seizure, hypotension, and cyanosis in the early stages of poisoning is associated with an increased risk of side effects and death and are serious warning signs. Early diagnosis and intervention can improve outcomes of methadone-poisoned children.

## Introduction

Methadone is a long-acting synthetic opiate, which has been used in morphine and heroin withdrawal programs since 1960 (1). The increasing use of methadone has increased methadone poisoning in children (2-4). Opiums General side-effects of Opiums, such as decreased consciousness, respiratory disorder, and secondary hypoxia, and seizure along with its long-acting effect, expose poisoned children to the risk of subsequent side-effects or death. Rare side effects, including acute cerebral ischemia due to cardiac complications and sudden death have been reported following methadone poisoning (2, 4-6). This research aimed to study the causes of incidence of methadone poisoning in children and its side-effects, and hereby to achieve fundamental solutions for early diagnosis, supportive measures, and reducing the cases of poisoning.

## Materials & Methods

All methadone-poisoned children referred to Hamadan’s Be’sat Hospital (A Hamedan Pediatric Center) from June 2007 to March 2013 were studied in this research. In patients included in this study, definitive history of poisoning was expressed by their parents, and in case of suspicious history or denial by parents, methadone presence was confirmed by urinalysis. In our hospital, the treatment protocol for methadone-poisoned patients includes initial washing, administration of activated charcoal, and injection of naloxan (10-100 mcg/kg/hr) for three days for all patients and also diagnostic and supportive measures depending on the case. Patient’s information, including personal information, poisoning substance, early symptoms, duration of hospitalization, need for mechanical ventilation and acute and chronic side-effects was extracted from their hospital records and entered into data collection forms.

## Results

**Fig 1:**
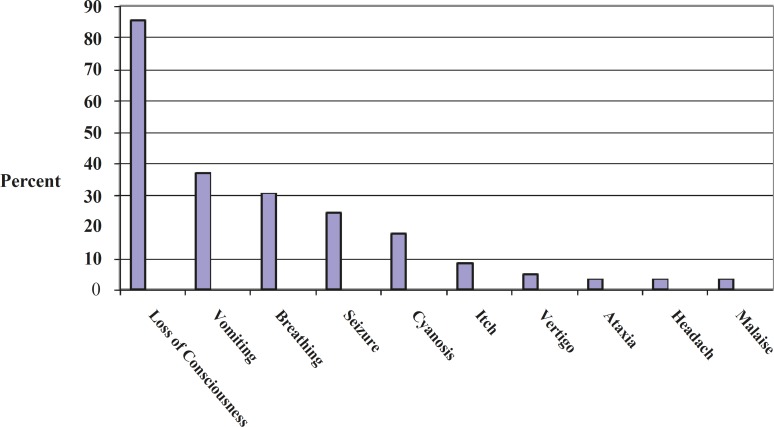
MRI Shows Acute hydrocephaly and interstitial edema (A) and T2 hyperintensity in cerebellar hemispheres (B)

**Diagram 1:**
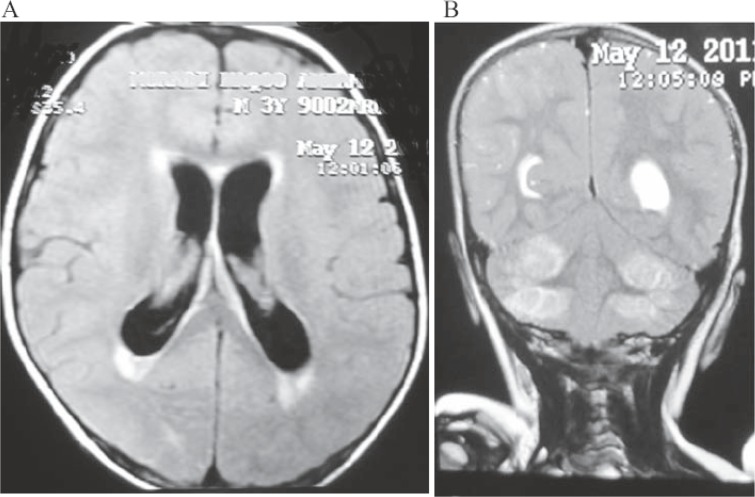
The main compliants of the parents of children with methadone poisoning

In about 5 years, 62 methadone-poisoned patients were admitted to Be’sat hospital. The mean age of the patients was 53.24±29.5 months (the youngest and oldest patients were 1 and 120 months old, respectively); 54.8% and 45.2% of the patients were male and female, respectively. Only one patient had a history of developmental delay and the rest were developmentally and mentally normal. The mean time between drug use and referral to hospital was 3.45±2.85 hours. There was a significant relationship between delayed referral to hospital and increased bradypnea (p=0.007). Also, 25.8% and 58.1% of patients had taken methadone tablet and methadone syrup, respectively. Methadone form was unknown for the rest of patients. In 30.6% and 51.6% of cases, methadone was mistakenly given to children by parents and taken by children themselves, respectively. In one case, the drug was intentionally administered to the patient. Cause of poisoning in the rest was unknown. In 80.6% of cases, parents mentioned poisoning in their first description. Urinalysis confirmed poisoning in other cases, in whom poisoning was denied by parents or of which they were unaware. The most common initial complaint mentioned by parents was decreased consciousness (85.5%) (Diagram 1). In 38.7% and 46.8% of patients, drowsiness and decreased consciousness with higher levels of drowsiness was observed, respectively. Seizure was experienced by 24.4% of patients. The most common finding during examinations by doctors of the hospital was decreased consciousness (91.9%). Decreased consciousness lasted more than 24 hours in 8 patients. In two cases, it lasted more than 5 days, both of which resulted in brain death. The mean time for returning to full consciousness was 18.50±86.60 hours. Miotic pupil and respiratory depression was observed in 82.3% and 69.4% of patients, respectively. Hypotension was observed in 6.5% of patients. The mean hospitalization duration (except for PICU hospitalization days) was 2.27±1.50 days. Due to severe symptoms, 36 patients were hospitalized in PICU which lasted 3.69±1.63 days. There is a significant relationship between seizure (p=0.005) and early signs of cyanosis (p=0.007). Mechanical ventilation was needed in 9 patients, which lasted 14.68±86.97. There was a significant relationship between mechanical ventilation and seizure (p=0.001) and early symptoms of emesis. Brain imaging (MRI or CT scan) was requested for 14 patients due to unusual symptoms and disorder, which indicated disorder only in 3 cases, one case of cerebellar edema and hydrocephalus (Fig1) and two cases of generalized cerebral edema. Aspiration pneumonia occurred in two patients during hospitalization. Cerebellar ischemia and secondarily obstructive hydrocephalus was observed in one case. Ventriculostomy was done on the patient and consequently, symptoms were improved after a few days. In one case, the patient showed symptoms of serum sickness and arthralgia 10 days after being discharged from hospital, which were gradually improved. Also, 59 patients with full recovery and one with relative side-effects (transient hydrocephalus) were discharged. There were 2 cases of death (3.2%), both of which were secondary to prolonged hypoxia and brain death.

## Discussion

Considering the fact that methadone poisoning is one of the most important causes of poisoning in children in our society, awareness of its early symptoms and secondary side-effects can help for early diagnosis and improvement of patient prognosis. The results of our study on the most common early symptoms of methadone poisoning are similar to those of previous studies. Decreased consciousness, respiratory disorder, cyanosis, and seizure were the most common complaints by parents at the time of referral to our hospital. In a study by Zamani et al. (2010) on opiate poisoning in children, side-effects included miosis (90%), decreased consciousness (88.4%), respiratory depression (28.4%), and seizure (10.3%) (7). In our study There may be a relationship between greater incidence of seizure and respiratory depression and the severity of symptoms in methadone poisoning compared to other opiates. In a study by Farnaghi et al. (2012) on methadone-poisoned children admitted to Loghman hospital, drowsiness, miotic pupil, emesis, shallow and slow breath, and apnea were observed in 75%, 68%, 61%, 57%, and 40% of cases, respectively (7). Aspiration pneumonia occurred in 17% of patients, which were statistically significant (p<0.022). There are a lot of similarities between the results of that study and those of our study. 

Cerebellar ischemia and secondary hydrocephalus were observed in one patient. Another similar case has been reported in the United States (5). Our patient went through a good clinical course but the one in the U.S. died of brain death. The mechanism of development of transient ischemia in the posterior regions of the brain and cerebellum and the mechanism of the developing cytotoxic edema by opiums via stimulation of μ receptors by these substances can explain the above mentioned phenomenon (2,8,9,10). The present study indicated that seizure, hypotension, and cyanosis in the early stages of poisoning are acute warning symptoms of an increased risk of death and side-effects. There may be a significant relationship between the need for ventilator in the case of emesis and aspiration pneumonia. This fact indicates the necessity of emesis management. Although our study did not show a significant relationship between delayed referral to hospital and prognosis, which indicated that delayed referral can be associated with increased respiratory disorder.

In Iranian addiction centers, methadone syrup is delivered to addicts in containers other than factory containers, which increases the risk of mistakenly use by adults and children. Many children had used methadone syrup instead of water or soft drinks. In the majority of cases, to whom methadone had been mistakenly given by their mother, she had mistakenly used it instead of antipyretic or cold syrup. These cases can be decreased by providing containers with child protective cap and hazard labele. Methadone tablet may be a more reliable method to decrease poisoning cases in children. 

**In conclusion, **preventive measures against poisoning with opiates, especially methadone in children are of great importance. The possibility of opiate poisoning should be considered in children with symptoms, such as decreased consciousness, respiratory depression, cyanosis, and seizure. Early diagnosis and intervention can improve outcomes in methadone-poisoned children. 
